# A Novel AI-Based Visual Stimuli Generation Approach for Environment Concept Design

**DOI:** 10.1155/2022/8015492

**Published:** 2022-06-24

**Authors:** Yingjing Duan, Jie Zhang

**Affiliations:** School of Design, Jiangnan University, Wuxi, China

## Abstract

The nature of environment concept design is a visual-based issue, where designers need to find loads of visual stimuli to create the high-quality concept. This paper aims to introduce a novel AI-based method to automatically generate images as design inspiration for environment concept design. Through interviewing eight professionals, we discovered that acquiring the design stimulus with inspired “composition” in a short time is the prioritized need of designers. This paper takes spectacular ambience for example. Through testing six classic GAN model variants trained by a self-made data set, we selected qualified four models to generate black and white thumbnails as stimuli with spectacular ambience. Moreover, we conducted a qualitative study of the outputs of the four models in a manner that invite eight designers to discuss. Finally, we summarized five key factors that influence designers' satisfaction with generated visual stimuli and discuss future directions that are worth studying.

## 1. Introduction

Conceptual design is an early stage of the design process, which involves information gathering, idea generation, and concept evaluation via a collection of sketches, images, and written statements to define design direction, as well as plays an instructive role in the following process of the produced objects design [1]. With the development of digital technology, the entertainment industry system, such as games and film, tends to mature, and the concept design that is originally widely used in product and architectural design begins to appear in the early stages of film and TV shows production, and its role is to visually present various settings in the planning and provide guidance during mid- and post-design phases, such as modeling, scene construction, or prop design. Environment concept design is a key part of digital industrial production. It requires designers to visually present scenes that conform to the worldview or plot development through the study of style, color, ambience, composition, and other knowledge about design and aesthetics, whose essence is visually based design. As shown by Gabriela Goldschmidt research [2], when designers are required to solve design problems at a conceptual level, especially visual-based design issues, the presence of visual stimuli has an effect on the qualities of the solutions they arrive at. Therefore, in the early stages of concept scene design, designers need to look for a large number of visual stimuli to support concept expansion.

With the advancement of artificial intelligence, its techniques have been applied in many domains and gradually become significant assistance to help the development of other fields. In design, artificial intelligence boosts the efficiency of designers substantially via its powerful capabilities of data mining and analysis [3]. In design ideation, a variety of AI-based methods are proposed, which not only can create a larger number of cross-domain idea associations but also can advance the ideation process quickly and easily in terms of quantity and novelty [4]. Although the creativity supporting method for design ideation has yielded a wealth of research results, these studies are mostly in the areas of product, apparel, or graphic design, but not in the area of environment concept design. Therefore, the purpose of this paper is to present an AI-based method, which can generate loads of visual stimuli randomly for expansion of design solution space to increase the quantity and diversity of generated design concepts in the ideation of environment concept design.

The rest of the paper is structured as follows. The next section reviews the literature regarding the influence of visual stimuli on design ideation and the proposed AI-based methods for design ideation. In the following section, we interviewed eight design professionals to identify the challenges in ideation as well as the potential solutions. Then, we introduce our method in detail and discuss the results. Recommendations for further studies and concluding remarks are finally made.

## 2. Related Work

### 2.1. The Influence of Visual Stimuli on Design Ideation

Ideation is an essential step in the integral design process to generate, develop, and communicate ideas, where the idea is a basic element of thought that can be either visual, concrete, or abstract [5]. In visual-based design, image materials as design stimuli have a positive effect on the quality, novelty, and diversity of design outputs. Many studies have been carried out to understand the effects of image inspiration in design ideation.

In terms of the richness of visual stimuli, an empirical study has been conducted by Goldschmidt and Smolkov [6] who have revealed that the presence of visual stimuli of different kinds can aﬀect performance. In this study, the designers were exposed to different situations (e.g., no visual stimuli, sketches as stimuli, and rich and diverse stimuli), whose performances were measured in terms of practicality, originality, and creativity scores in light of outputs. Besides, this study also has indicated that designers tend to be particularly sensitive to various types of external stimuli, especially surrounding visual displays. Cardoso and Schaub [7] have carried out a research study to expand on the type of visual stimuli (e.g., line-drawing and photographic). The result shows only one type of representation of external material could induce high levels of attribute repetition, and diverse and rich visual stimuli could reduce design fixation.

Regarding ambiguity, Tseng [8] has conducted an experiment to explore the influence of visual ambiguity on design ideation, where the ambiguous images used as stimulus were classiﬁed into high, moderate, and low ambiguity and subjects were asked to use the ideas suggested by the visual cues to design a novel table. The results have indicated with higher ambiguity of visual display can provide the designer with greater freedom to search for more diverse ways to resolve presented uncertainties, thereby increasing novelty during idea generation. Through an experiment to investigate the effect of ambiguous visual stimuli on creativity in design ideation, Jang et al. [9] have presented that ambiguous stimuli from dissimilar concept pairs fostered higher elaboration in individual working.

In addition, Laing and Masoodian [10] have carried out a study with 18 graphic design students, who were provided with images related to the aesthetic tastes of clients and their market competitors. The findings demonstrate that the differences in creativity are minimal measurably between the two types of images, but the interview data from participants have shown that pictorial inspiration has a positive effect on the way to approach the tasks and increases their satisfaction with the textual task descriptions provided.

In summary, the studies motioned above have three main effects on design ideation:

1. Rich and diverse visual stimuli have a positive effect on the phase of conceptual expansion in design
2. Availability of visual display can provide designers with benefits to their experience during the ideation phase to improve efficiency
3. Higher ambiguity of figures used as design cues could expand the freedom of designers for discovering more innovative ways to increase the novelty of generated ideas and reduce fixation

Our study is based on the conclusions the existing literature has suggested and then to obtain more precise needs about concept generation in environment concept design by interviewing design professionals. We set the goals to be achieved by our method eventually.

### 2.2. AI-Based Approaches for Design Ideation

As a brunch of computational creativity, a variety of AI-based methods for promoting creative idea generation for design have been proposed with the advancement of computational creativity, which can be categorized into stimuli retrieval and stimuli generation accordingly to the ways they are produced [11].

In terms of retrieval, Hao et al. [12] have introduced an evolutionary computation method, which automatically generates language terms as design stimuli through retrieving from a vocabulary base based on 50,0000 granted patents. Shi et al. [13] have proposed an unsupervised learning ontology network for design information retrieval, focusing on the associations between design and engineering by data-driven text mining and semantic network analysis. Sarica et al. [14], through employing the latest natural language processing techniques to extract semantic-level knowledge in all technology fields from six million US patent documents, have introduced a methodology named TechNet to retrieve the engineering knowledge in a field and explore engineering concepts around the field for future design considerations and innovation.

With the development of AI image generation technique, the computer can generate imagery material directly for creativity support. Chen et al. [4] have proposed a visual stimulus generation model, whose generated images visually captured partial elements of two distinct concepts in two different domains. Li et al. [15] have introduced a product concept generation method, which was formed by an affective recognition model to mark the affective preferences of users and product design GAN model (PD-GAN) to generate conceptual images with affective preferences via deep learning techniques and Kansei engineering. Compared to retrieval, there are relatively few studies about a generation now. Empirically, as the generation model can have a variety of new images, it has the infinite potentiality to be employed during the ideation phase in design.

The above literature review shows the crucial technique employed for approaches to AI-based design ideation is semantic network. Research studies start off by constructing a design knowledge base, which is formed by an enormous number of existing excellent design cases or patents, then retrieve the knowledge base through AI techniques, and generate semantic-level stimuli by analogizing, filtering, combining, and classifying concepts in the knowledge base. As to visual representation, the process of stimulus generation requires semantic understanding first before visual display generation. This type of method is suitable for well-defined design problems with concrete requirements, but not for ill-defined ones with abstract requirements, such as issues related to environment conceptual design. Therefore, this paper intends to develop an AI-based approach to solve this problem.

## 3. Requirements Research

In this section, we will interview eight design professionals in the domain. By analysis of the results of the interview, we then set the goals of our approach.

### 3.1. Interviews with Design Professionals

Design professionals (five males and three females), who are invited to participate in the interview, are all majored in environment concept design; four of them are graduate students; and the other four work in the company, who all have over five years of experience in this domain. The interviews were conducted online via screen-sharable video software. Each interview took about 60 minutes. Two researchers as interviewers carried out and recorded the interviews. Besides, for later analysis, we video-recorded the interviews.

In the interview, we mainly asked about: (1) their ideation process in environment concept design practice, (2) barriers and challenges that trouble them during the phase of concept generation, and (3) without warring about limitations, their ideal solutions to address these challenges. During the interviews, the researchers pursued further questions about the ambiguous answers given by the interviewees so that they could get clearer and more enlightening answers.

### 3.2. Results

Afterwards, we arranged the feedback from the professionals and summarized them in three aspects: ideation process, challenges in ideation, and ideal solutions as follows.

#### 3.2.1. Ideation Process

We identified three steps of the environment concept design ideation process:

*(1) Ambience Defining*. Designers analyze the design requirement from the scenario to define the ambience in the conceptual environment. Ambience refers to the feeling and emotion that the scene wants to convey, which is usually defined by the semantics of composition color and elements on the visual guiding line in a picture. The definition of ambience requires the designer to analyze the design requirements given by the plan, extract, or derive the description related to the ambience, which is the key part in the early stage of ideation and provides guidance for the next steps.

*(2) Composition Setting*. The expression of the ambience is entwined with the composition in environment design. Composition is combinations and arrangements of different elements in a conceptual environment graphic. Generally, during this phase, designers first select the composition category; then determine the position of subjects, such as landmark buildings or other objects in the visual center of the picture; and finally arrange or combine the other elements by designing a visual guideline, which is used to direct the viewer's gaze to the center of vision. Composition is the beginning of the concept visualization. In this stage, experienced designers usually analyze the planning scheme, combine the experience and aesthetic principles to create mental images, and then look for visual materials that can be employed as stimuli to find the optimal solution according to the mental images, while novices usually need to collect a large number of design stimuli before they produce creative imagery.

*(3) Hue Setting*. Color is another essential element that embodies the ambience. Color refers to the large blocks of color in a picture. The hue is composed of two main parts: color matching and color proportion. Color matching usually depends on emotions, the climate, time period, and geographical location in environment concept design, and color promotion is mainly affected by the composition. Therefore, the hue setting depends not only on the ambience but also on the composition. In this part, as with the composition setting, designers also need to look for a large number of visual stimuli to support the idea generation of hue.

**Figure 1:**
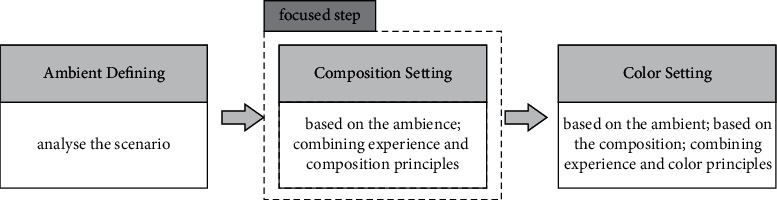
The process of ideation.

Overall, the environment concept design is mainly composed of three stages, and its process is shown in Figure 1. We find that the ideation of composition is a key part of the phase of ideation in environment concept design, which plays a role in carrying forward the upper and lower levels. The setting of composition is not only a preliminary presentation of the ambience in a conceptual environment but also influence the setting of the hue. Therefore, our supporting creation method focuses on the assistance of the composition. The reporting and analysis of the interview results of the following two aspects will also focus on the composition.

#### 3.2.2. Challenges in Ideation

We also identified design professionals' thoughts on hinders that interfere with concept expansion in design ideation. Through interviews, we learned that compositional creative thinking is primarily by two aspects, namely composition principles and the designer's experience. Composition principles here mainly refer to the correspondence between composition and ambience; a certain type of ambience is most appropriately presented by corresponding compositions. The nature of composition principles is knowledge, which is obtained by the designer during their learning period. Another impact is previous experience, involving not only designers' own experience but also the experience of others. The main presentation of previous experience is visual material, such as completed graphics, photo references, and video clips, in which ambience is similar to the design output required to present.

Regarding the hints encountered in the ideation process, designers generally think that there are two types of challenges. One barrier is that the incomplete mastery of composition principles leads to barriers in the process of generating mental imagery in the early stage of conception ideation, often when the designer is a novice. Another one is that designers cannot quickly and accurately find materials that meet their needs to stimulate their creation effectively, through existing methods. Designers often use the engine to search for keywords to collect visual material. However, such an approach is usually suitable for the retrieval of elements with specific semantics. As the composition is an abstract word, the search engine cannot give the designer a satisfactory stimulus in the semantic-based search process, which makes it difficult for designers to collect the visual material closely related to the composition.

#### 3.2.3. Ideal Solution

In response to the two challenges, the designers explain their own approach to overcoming these challenges and imagined the ideal solution without considering the constraints. For the first challenge about mental imagery, designers believe that in addition to a more thorough grasp of the aesthetic knowledge related to composition, they tend to also collect a large number of visual displays with similar features to mental imagery, such as black and white thumbnails or digital speed paintings. These materials are visual presentations of others' early ideas, although they do not contain rich details, but their general structures are clear, and their ambiguity provides designers with space for exploration. Therefore, when it comes to the desired solution, designers said that they hope that the computer can automatically generate mental image pictures based on composition principles to help them successfully pass through the early stage of ideation.

For another challenge related to collecting effective visual stimuli, designers explained that when they have a clear mental image, they will try to summarize keywords that accurately describe intentions and search for them. Usually, designers expand the scope of keywords to expand the search scope of search engines. In terms of the ideal solution, the following two ideal solutions have been repeatedly proposed by more than half of the designers, one is to hope that by inputting the composition sketch, the computer can automatically match the complete design case similar to the composition of inputs; the other is to hope that by inputting some composition types, the computer can output materials diversely with similar composition.

Overall, designers have both semantic thinking about design issues and aesthetic experience based on visual thinking during the phase of ideation in environment concept design. Effective visual stimulation not only assists the designer to produce the appropriate mantel imagery but also promotes the design process after designers create creative imagery, which leads designers to find the optimal design solution.

## 4. The AI-Based Approach

**Figure 2:**
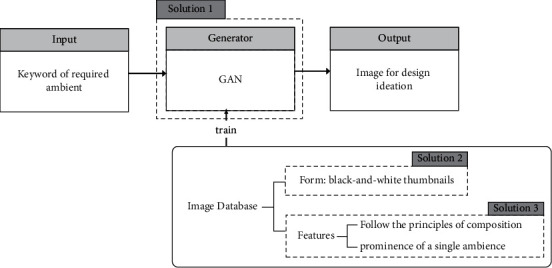
The framework of our approach.

Based on the interview results in the last section and research findings in related work, we proposed three main goals of the AI-based method we introduced:

Goal 1: this method can quickly generate visual stimuli for the composition according to the needs of designers, which are diverse and numerous; goal 2: the visual stimuli generated by this method need to follow composition principles; and goal 3: the visual stimuli generated by this method need to be similar to a mental image. The framework of our approach is shown in Figure 2.

In goal 1, the generator is responsible for stimulus outputs, and its essence is image generation. Compared to other image generation methods, GAN [16] is able to generate images quickly with high quality. Thus, we will select the appropriate GAN variant models. Our requirement for the generation model is that the trained model can generate a great number of diverse images as visual stimuli in a short time. For the achievement of goal 2, we will train the model on a training set that follows the composition principles and had a single ambience feature to generate visual stimulus. In goal 3, to make the generated images similar to the mental imagery, which is ambiguous but clear in structure in general, we set the picture type of the data set to black and white thumbnail sketches. We then take the generation of visual materials with spectacular ambience as an example to introduce our method in respect of data collection and generator implementation in detail.

### 4.1. Data Collection

The data used to train the model are from the Google Image search engine, and the training set consists of 153 black and white thumbnails. The reason why we chose thumbnails is that through interviews, thumbnails are generally considered by designers to be a visual presentation of mental images and the characteristics of overall structure clarity that allow designers to quickly obtain effective information from it. Meanwhile, due to the ambiguity brought about by its lack of details, designers are provided with a vast creative idea space, so this is an effective form of design stimulation. In addition, the black and white feature is to exclude the interference of color, as color is another major factor affecting the ambience, and the computer improves the quality of the generated images by extracting a single data feature. Since taking spectacular ambience as an example, the images that make up the data set need to have prominent features of spectacular ambience.

**Figure 3:**
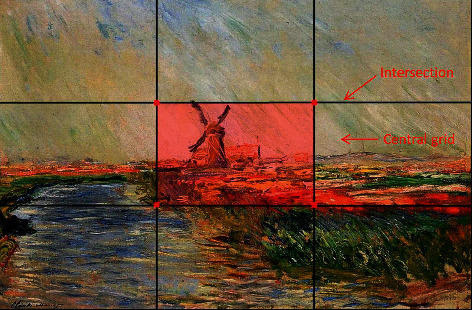
Composition of the rule of thirds.

We summarized the characteristics of the composition: the cases that embody this ambience are mostly composed in the rule of thirds by learning the methods of environment concept design and studying the excellent cases recommended by designers with spectacular ambience. The guideline proposes that an image should be imagined as divided into nine equal parts by two equally spaced horizontal lines and two equally spaced vertical lines and that important compositional elements should be placed central grid or intersections of golden section lines [17], as shown in Figure 3. Generally, this composition not only can highlight the theme elements but also can increase the depth of the scene space through the relative blank picture outside the central grid to create a spectacular view. In addition, the themes of design cases with the ambience are mostly the surroundings of large buildings such as palaces and castles or natural landscapes such as mountain views.

**Figure 4:**
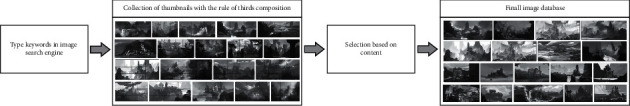
The process of making the data set.

After the study, we start to make a data set based on the compositional features. First of all, we searched on the Google image engine by inputting the keyword—conceptual environmental thumbnail sketches—and then browsed the images retrieved by the engine one by one, analyzed the composition method, and downloaded the cases that follow the rule of thirds. We obtained a total of 209 images during the period. Subsequently, we invited 3 professional designers to identify the ambience of these cases with us to form the data set for training. The process is shown in Figure 4.

### 4.2. Implementation of Generator

This section introduces the process of selecting a generator in detail and discusses the results of the outputs. In order to find suitable GAN models, this paper uses the prepared data set to train different GAN models, and then the researchers test the results from both duration for generation and diversity of outputs. The results of the test will determine whether the model can enter the next qualitative research. The qualitative study is conducted in a group discussion with design experts invited to participate in the interview in the third part. We analyze the results of the interview and draw a conclusion eventually.

### 4.3. Training and Testing of Different Models

During the next phase, we tested both the generation duration and the diversity of outputs of each model.

A generative adversarial network (GAN) is a kind of method that can learn the deep representation of data without a large amount of annotation training data sets. By updating two networks through a backpropagation algorithm, the data with established characteristics can be generated in a competitive way. Although GAN has obtained remarkable achievements in image generation tasks, the classic GAN network still has problems such as unstable training and less quality and diversity of generated images. Therefore, how to obtain a stable and high-quality generative adversarial model has always been the focus of research. Several representative networks are follows.

Compared with traditional GAN networks, DCGAN [18] networks put forward a series of architectures to combine CNN with the original GAN network effectively, which improved the depth of the feature extraction network and the quality of generated images. WGAN [19] proposed a generative adversarial model based on Wasserstein distance, which not only solved the problem of training instability but also provided a reliable indicator of training to improve the quality of generated samples. LSGAN [20] adopted the same idea as WGAN. Specifically, the loss function of LSGAN was changed from cross-entropy loss to the least square loss function to obtain images with higher quality and diversity. WGAN-gp [21] focused on the training instability caused by the weight clipping strategy in WGAN. A method of gradient penalty was proposed to solve the above problem and make the training of WGAN more stable. For the effect of the local equilibrium state in training, DRAGAN [22] proposed a novel gradient penalty scheme, which effectively improved the stability of GAN. Besides, a novel progressive GAN training model is proposed in PGGAN [23], which solved the problem of high cost in generating high-resolution images under the original GAN framework and greatly improved the quality of generated high-resolution images.

In summary, based on the self-made data sets, we have tested the above six GAN variant networks with certain advantages in model stability, generated image quality, and diversity, including DCGAN, WGAN, LSGAN, WGAN-GP, DRAGAN, and PGGAN. Then we analyzed the generated results in detail. The characteristics, corresponding advantages, and training parameters of the six models are shown in Table 1.

#### 4.3.1. Generation Duration Test

**Figure 5:**
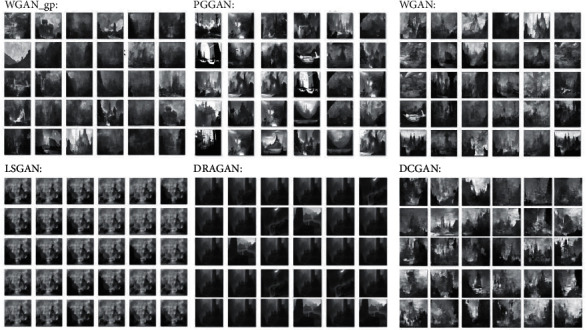
Some outputs of six models.

We ask each of the six trained models to generate 100 images, and some of the outputs are shown in Figure 5. The mean and standard deviation (in second) of each model are shown in Table 2. Among them, the average generation duration of WGAN models is the shortest and most stable. The passing criteria for this testing are as follows: the generation duration of a model is less than or equal to 10 seconds. Otherwise, it will be eliminated because it does not meet the requirements of goal 1—quick generation of numerous visual stimuli. The results reveal that the generation duration of each six models is less than 10 seconds, so they all pass the test.

#### 4.3.2. Diversity Test

In this test, we calculate the diversity rate for 100 outputs of each model in the generation duration test, formula as follows:

(1) $$\begin{array}{c} \text{Diversity rate}=\frac{\text{The number of filtered images}}{\text{The total number of image}}. \end{array}$$

**Figure 6:**
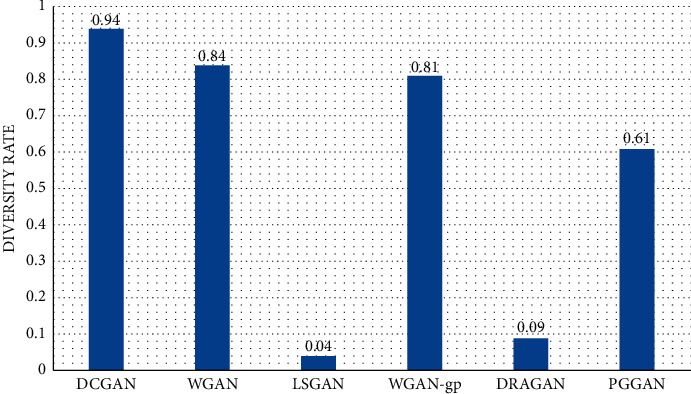
Diversity rate of models.

In the process of filtering images, we regard two or more pictures that are similar or identical to each other as one and delete the extra pictures to finally get the total number of pictures after filtering. The criterion for judging whether pictures are similar is that the black and white blocks of two or more pictures are distributed consistently together with the same spatial level. The criterion for passing the test is that the diversity rate is higher than or equal to 60%; otherwise, it will be eliminated as it does not meet the requirement in goal 1, generation of diverse visual stimuli. The diversity test results are shown in Figure 6, from which it can be seen that the diversity of LSGAN and DRAGAN is less than 60%, so they are eliminated and cannot enter the quantitative analysis stage. As for the causes for the low diversity, we speculate that it may be because the data set is not rigorously de-reprocessed, where the images with high similarity are removed, resulting in the overfitting of the models.

#### 4.3.3. Qualitative Research

The qualitative study is conducted in a group discussion with eight design professionals who participated in the expert interviews before. The reason why the format of the group discussion is because we hope that designers have sufficient freedom to express their views under the given topics, which can help us obtain the advantages and disadvantages of the designers' analysis of generated images of the four models, so as to summarize the designers' concerns and requirements for AI-generated visual stimuli.

**Figure 7:**
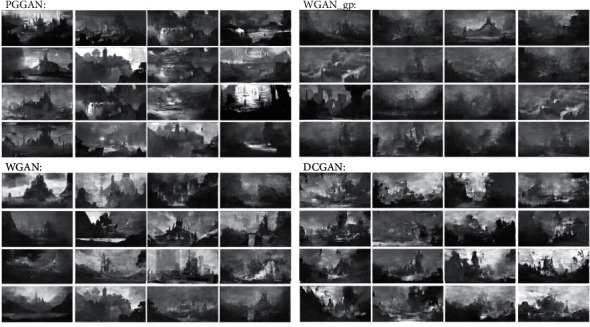
Some of the generated images as stimuli.

The interview process is as follows: we describe the design requirement—designing a spectacular environment for the surroundings of a large building, to the participants in detail and give them five minutes for composition ideation. Then, we present the 100 resized images as design stimulus generated by the four models in advance to the designers, and some of the images are shown in Figure 7. There are two topics in the discussion: (1) analysis of advantages and disadvantages of the generation results of each four models and (2) horizontal comparison of the generation results of each four models, and finally, they have to form a unified opinion. Discussions take place online, using online conferencing software with video and audio access for an unlimited period of time. The discussion is recorded by two researchers, and at the end of the discussion, the researchers confirm the advantages and disadvantages of the four models' outputs and the uniform results of the satisfaction ranking with designers and then obtain the final results.

The advantages and disadvantages of the outputs are shown in Table 3.

The designers' satisfaction with the outputs of the four models is sorted from highest to lowest: WGAN and PGGAN, WGAN-pg, and DCGAN. In general, in terms of coherence and completeness, there is no fragmented or split picture in the results of the four models, so it is basically in line with expectations. Besides, based on the results of the discussion, we can find that satisfaction with the generated stimuli of different models is mainly affected by spatial levels, the recognizability of main objects, the diversity of the arrangement of elements, and their responsiveness to composition principles. These factors are weighted equally by designers, so satisfaction with the results of model generation depends on how well the model responds positively to these factors. In addition, the designers claim that the visual stimuli generated by the four models are basically a response to the design requirement.

#### 4.3.4. Results Analysis

We summarize five factors mentioned above that affect the quality of generated visual stimuli—completeness, spatial levels, recognizability of main objects, diversity of the arrangement of elements, and responsiveness to composition principles, which can also be regarded as attributes of generated visual stimuli that are the focus of designers. This paper will explain these five as follows:

- Completeness: In environment concept design, the composition emphasizes integrity; it is the preliminary conception of the layout and organization relationship of elements. Therefore, completeness directly affects the designer's initial impression of the visual stimulus, and the highly complete image can clearly present the distribution of the elements, attracting the designer to further observe.
- Spatial levels: The essence of environment design is the design of space, so designers need to consider not only the elements in the conception but also the way the elements are presented in space, which is a process of space planning. In general, an excellent scene design presents at least three levels of space, namely close-up, medium-shot, and far-range. In the stimulus generated by the model used in this paper, the spatial level is mainly reflected in the difference between the black, white, and gray levels. WGAN-pg is because the gap between black, white, and gray levels is not large, resulting in the space levels are not rich enough, only two layers or even one layer, thus becoming the object of criticism by designers.
- Recognizability of main objects: The main object occupies the visual center of the picture, which is the most eye-catching part and the area with the most detail in an image. Therefore, designers tend to define the position of elements initially, and then design subjects. In the generated visual stimulus, the recognition of main objects depends on the details of the outline of the subject and the difference with the surrounding elements in the black and white level. Thus, high recognizability can help designers successfully generate ideas about the subjects.
- Diversity of the arrangement of elements: Diversity refers to the diversity of the arrangement and combination of elements under the premise of following the principle of composition. In this paper, the generated visual stimuli should follow the principle of nine-square grid composition, which stipulates the position and proportion of main objects. But the proportion, arrangement, and combination of other elements are free, which is also the embodiment of the diversity of visual stimulus composition.
- Responsiveness to composition principles: It refers to whether the picture follows the composition principle or to what extent it follows the composition principle, which is an important criterion for designers to judge whether the visual stimulus meets its aesthetic standards.

In addition, we also find that the ambiguity of the elements needs to be controlled within a certain range. However, existing studies of visual stimuli have shown that the ambiguity of stimuli can stimulate designers' creativity by expanding the space for the exploration of design solutions. However, in the environment concept design, excessive ambiguity not only cannot stimulate the designer's creative thinking but also will become an obstacle in the ideation process. For example, the images generated by DCGAN because of the high degree of ambiguity of the elements lead designers to claim that the model is flawed.

Overall, this paper realizes goals 2 and 3 by making a model training data set that meets the requirements of designers and achieves goal 1 by testing different GAN variants to select appropriate ones as the generator. In the qualitative analysis of the results of GAN variant generation, WGAN and PGGAN were considered by the designers to be the most suitable models for generating visual stimuli. Although the method proposed in this paper has successfully achieved the stated goals, there are still shortcomings. The problem is that the composition form of visual stimulation under a certain ambience is relatively single, which is easy to trigger design fixation. The main reason for this problem is that this paper employs a “one-to-one” method when making the data set, which means one ambience corresponds to a fixed composition, such as the spectacular ambience corresponds to the composition in the rule of thirds. Therefore, the process to make the data set is to first filter the composition and then check whether it corresponds to the ambience. However, when actually solving the problem of environment concept design, there is a diverse correspondence between ambience and composition principles. In the case used in this paper, for example, the spectacular ambience can also be expressed through other compositions or atypical compositions in the rule of thirds. Limiting the presentation of the ambience to an exclusive composition is bound to reduce the diversity of composition. Therefore, exploring the correspondence between ambience and composition principles and finding a more suitable data set production process are the problems that need to be further solved in this paper.

## 5. Conclusion

This section will discuss the expansion of AI-based approaches for design ideation in two aspects, forms and the process of methods, as well as make concluding remarks.

### 5.1. Forms

As far as the existing method is concerned, according to the form of the stimulus produced, it can be divided into textual materials and visual materials. Based on the process of producing stimuli, it can be categorized into retrieval stimuli and generative stimuli. Thus, there should be four types of stimuli, namely retrieval textual stimuli, retrieval visual stimuli, generated textual stimuli, and generated visual stimuli. Producing retrieval stimuli has always been researching hotspot, while there are relatively few methods for generated stimuli. However, with the continuous development of the generative model, the efficiency of the generative model is getting higher and higher, and the tasks that can be finished by the generative model are becoming more and more complicated. Therefore, it is worth exploring the potential of generative models and making them do their best to support the creative thinking of designers. Additionally, producing more novel forms of design stimuli by AI, such as sound, is also a direction in further study.

In addition, both retrieval stimuli and generated stimuli have their own advantages and disadvantages. The advantage of the retrieval stimulus is that the quality is stable; the disadvantage is that retrieval stimulus usually roots from existing cases and cannot be adjusted according to the requirements of the designer, so the plasticity is not strong. On the contrary, the advantage of generative stimuli is that they are highly malleable, and designers can adjust the parameters of generative stimuli according to their own needs, but the quality of generative stimuli is unstable, and low-quality design stimuli will become an obstacle in the design ideation. Therefore, a future study should focus on how to integrate the two types of design stimuli in a way that fully exploits their respective advantages while avoiding the negative impacts of doing so.

### 5.2. The Process of Methods

**Figure 8:**
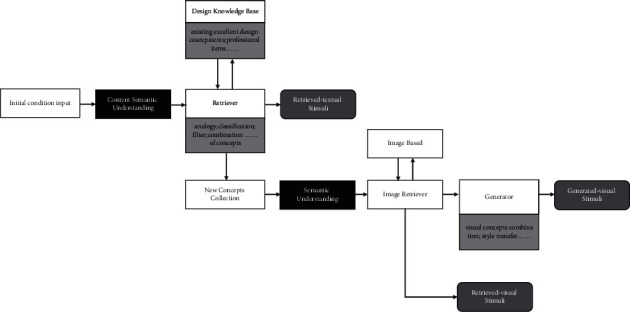
The general process of the existing method for producing design stimulus.

This paper summarizes the production methods of existing stimuli, as shown in Figure 8. It can be seen that producing the four forms of stimuli must be supported by semantic-level understanding and semantic knowledge base in the existing methods. This is because the semantic area in the brain plays an important role in creative thinking. Especially, when engaged in conceptual expansion, the brain's semantic processing network operates on overdrive, particularly in the higher-order regions that mediate lexical selection, controlled retrieval, combination, and integration processes [24]. In the field of artificial intelligence, with the development and improvement of NLP and computing creativity, computers can simulate the function of the brain's semantic network through algorithms, assisting designers in concept expansion. This is the reason why existing methods are mostly based on semantic networks.

However, conceptual extensions guided by visual thinking are also a key part of solving design problems. After the qualitative research of the fourth part, we find that in the composition ideation, the five factors that affect the effectiveness of generative visual stimulus are derived from the aesthetic evaluation of the picture in the field of visual art. Thus, we claim that when visual-based design problems, effective design stimulus, in addition to semantically supporting the expansion of concepts, also needs to bring a good aesthetic experience to designers visually. It can be seen that in the method development, researchers should conduct a comprehensive analysis of the object served by the method and the problems it needs to solve, and the method of inspiring design ideas can be based on aesthetic knowledge in addition to the semantic level. Creative stimulation can solely effectively serve the designer's cognitive process in order to achieve the true sense of supporting design ideation.

In conclusion, conceptual expansion is a crucial part of design ideation, in which effective stimulus can inspire designers to find the optimal solution. In AI-based approaches to design ideation, AI can improve the effectiveness of design stimuli by producing materials that are more in line with the designer's needs. This paper starts from the visual-based design problem of environment concept design, identifies the challenges, and obtains ideal solutions of hints through expert interviews. Through the summary and induction of the interview results, this paper summarizes the three goals of the new method. Subsequently, this paper achieves the stated goals by making its own data sets and selecting the appropriate generation model. Subsequently, we quantitatively analyzed the outputs of the selected generative model through expert discussions and summarized the five main factors affecting the designer's satisfaction with the stimulus generated by AI. The key to supporting designers in their creative thinking by exposing them to stimuli is to improve the effectiveness of design stimuli, and the reasons that affect effectiveness are complex and changeable. Therefore, in order to improve the effectiveness of design stimulation, it is necessary to have a deep understanding of the process of designers to design and conceive, find the difficulties encountered by designers in design ideation, and find the entry point of artificial intelligence to solve the difficulties, in order to achieve the true sense of auxiliary creative ideas.

## Figures and Tables

**Table 1:** The characteristics, corresponding advantages, and training parameters of the six models.

Model	Feature of model	Corresponding advantages	Training parameter			
			Epoch	Learning rate	Optimizer	Total parameter (M)
DCGAN	Effective combination of GAN and CNN	(1) Improvement of the depth of the feature extraction network; (2) enhancement of image resolution	8 × 10^3^	2 × 10^−3^	Adam	12.33
WGAN	Wasserstein-distance-based GAN	(1) Improvement of training stability; (2) improvement of generated image quality	8 × 10^3^	2 × 10^−4^	Adam	23.36
LSGAN	Replacement from cross-entropy loss to least squares loss	(1) Mitigation of gradient disappearance problem; (2) improvement of generated image quality	8 × 10^3^	2 × 10^−4^	Adam	23.36
WGAN_gp	Introduction of the gradient penalty	Improvement of training stability	8 × 10^3^	2 × 10^−4^	Adam	23.36
DRAGAN	Introduction of a novel gradient penalty scheme	Improvement of stability	8 × 10^3^	2 × 10^−4^	Adam	23.36
PGGAN	Introduction of a novel progressive GAN training model	Solution of high loss problem for generating high-resolution images	8 × 10^5^	2 × 10^−3^	Adam	46.14

**Table 2:** Mean and standard deviation of generation duration.

Model	Mean	±	Standard deviation
WGAN	**1.8132**	±	**0.0224**
DRAGAN	**1.8728**	±	**0.0614**
LSGAN	**1.9011**	±	**0.0675**
WGAN_gp	**2.0201**	±	**0.0482**
DCGAN	**2.0358**	±	**0.5322**
PGGAN	**5.3076**	±	**0.0364**

**Table 3:** The advantages and disadvantages of the four model generation results.

Model	Strengths of model	Weakness of model
PGGAN	The spatial level of the picture is rich; the clarity is high; and the position of the center in the vision basically follows the principle of composition.	The contour line of the visual center is not clear, and its small distinction from the surrounding elements is easily misleading.
WGAN	The pictures have rich spatial levels, high definition, and clear contour lines of the subjects whose position follows the principles of composition so that it is easy to distinguish from other elements.	The distribution of elements in the picture is monotonous, and the diversity is inadequate.
WGAN_gp	The completeness of the pictures is high, and the position of the main objects basically follows the principle of composition.	There is lack of spatial level, small difference between the main object and other elements, as well as lack of clarity in the pictures.
DCGAN	The completeness of the pictures is high with rich spatial levels.	The elements of the picture are ambiguous and illegible, which are arranged in a haphazard manner that does not follow the principles of composition.

## Data Availability

The data sets used to support the findings of this study are available from the corresponding author upon request.
